# SITRUS: Semantic Infrastructure for Wireless Sensor Networks

**DOI:** 10.3390/s151127436

**Published:** 2015-10-29

**Authors:** Kalil A. Bispo, Nelson S. Rosa, Paulo R. F. Cunha

**Affiliations:** 1 Department of Computer Science (DCOMP), Federal University of Sergipe (UFS), Aracaju, SE 49100-000, Brazil; 2 Centre of Informatics (CIn), Federal University of Pernambuco (UFPE), Recife, PE 50740-560, Brazil; E-Mails: nsr@cin.ufpe.br (N.S.R.); prfc@cin.ufpe.br (P.R.F.C.)

**Keywords:** semantic infrastructure, wireless sensor networks, ontology and semantic web, power consumption, software reconfiguration

## Abstract

Wireless sensor networks (WSNs) are made up of nodes with limited resources, such as processing, bandwidth, memory and, most importantly, energy. For this reason, it is essential that WSNs always work to reduce the power consumption as much as possible in order to maximize its lifetime. In this context, this paper presents SITRUS (semantic infrastructure for wireless sensor networks), which aims to reduce the power consumption of WSN nodes using ontologies. SITRUS consists of two major parts: a message-oriented middleware responsible for both an oriented message communication service and a reconfiguration service; and a semantic information processing module whose purpose is to generate a semantic database that provides the basis to decide whether a WSN node needs to be reconfigurated or not. In order to evaluate the proposed solution, we carried out an experimental evaluation to assess the power consumption and memory usage of WSN applications built atop SITRUS.

## 1. Introduction

Wireless sensor networks (WSNs) consist of a set of nodes (dozens, hundreds or thousands) able to collect, process and transmit data from the environment to other nodes or base stations. WSNs have a great potential for distributed applications and have been very attractive for many application domains, such as environmental monitoring, human-centric applications, military applications, support for logistics, vehicle traffic monitoring and other countless context-aware applications [1].

WSNs nodes have limited resources, such as processing, bandwidth, battery, and so on. One important feature of WSNs is that they can spend the smallest amount of energy to work for as long as possible. The amount of WSN nodes in one area and the fact that WSN nodes are usually distributed in areas of difficult access make recharging batteries almost impossible [2]. Ideally, WSN nodes should be kept in operation modes that minimize the power consumption [3,4,5].

WSNs continually collect information and generate a large volume of data that is normally handled and processed by applications. Furthermore, power consumption must always be taken into consideration. As more data are generated, more energy is consumed, thus degrading the network status. Energy efficiency is not only related to the power consumption of a single WSN node, but also to the entire network consumption.

As shown in previous research [3], the transmission of one bit over the network can consume as much energy as running thousands of instructions, because the communication subsystem has a much higher power consumption than the computing subsystem [6]. For that reason, communication must be improved through pre-processing before data dissemination. Another important issue is the sensing subsystem. It may be a significant source of power consumption, and for this reason, data must be acquired only if necessary.

In this context, this paper presents SITRUS (semantic infrastructure for wireless sensor networks) that mainly aims to reduce the power consumption of WSNs through reconfiguring applications and sharing their data. SITRUS is made up by a message-oriented middleware and a semantic information processing outside the network, which aims to make decisions about the WSN nodes, network and environment, using ontology to semantically enrich and process all data from WSN.

Considering what is being proposed, this paper has the following unique contributions: (1) a semantic infrastructure for WSN, with an architecture that works with data from WSN and ontologies and promotes communication among different networks supporting the decision-making process and unambiguous queries; (2) a message-oriented middleware for WSNs to handle the heterogeneity of data and applications and to improve data communication; (3) a reconfiguration service of applications, WSNs and WSN nodes based on semantic information; and (4) a semantic database with all knowledge about the WSNs and WSN nodes in order to avoid unnecessary queries on WSNs and to help the reconfiguration service to make decisions.

The choice for an infrastructure like SITRUS is motivated by a few reasons: the whole reconfiguration process does not suffer from human intervention; different networks can promote the integration of different applications by sharing data for the same context using ontologies and processing outside of the WSNs; and override the syntactic constraints on the messages by data enrichment supported by ontologies.

The remainder of this paper is organized as follows. Section 2 introduces basic concepts about WSNs, ontology and the Semantic Web, middleware and software reconfiguration, which are necessary to understand the rest of the paper. Next, Section 3 presents SITRUS, including its middleware for the WSN and semantic information processing module. Section 4 presents the experimental evaluation focused on power consumption, including the methodology used and the results. Section 5 presents an overview of related works. Finally, Section 6 presents the conclusion and some future works.

## 2. Background

This section introduces concepts that are essential in order to understand our proposal, such as ontology and the Semantic Web, middleware and adopted WSN technologies, such as the operating system TinyOS and the programming language nesC.

### 2.1. Ontology and Semantic Web

WSNs generate a large volume of data, which have a natural heterogeneity. Sharing and data integration are major challenges in WSNs. To solve this sort of problem, the Semantic Web [7] provides a common framework that allows data to be shared and reused among applications. Thus, the idea of web applications is extended to an integrated data web, which can be effectively shared by users and can also be easily processed by machines.

The Semantic Web enables people to express, in a machine-processable form, the relationship between different datasets and their properties. Therefore, it promotes a semantic relationship between data, allows machines to automatically understand data and is, consequently, able to process and infer new data information from existing ones.

The concept of ontology is the most important to define the Semantic Web. Ontology can be interpreted as the set of entities with their relationships, constraints, axioms and vocabulary. An ontology defines a domain or, more formally, specifies a conceptualization about it [8]. It could be the explicit, formal and shared conceptualization of a knowledge field [9].

This conceptualization refers to an abstract model, with the relevant concepts identified, of a phenomenon in the world (e.g., WSNs). Explicit means that the set of concepts used and the applied restrictions are previously and explicitly defined. Formal refers to the fact that an ontology is expected to be processable by a computer, which excludes definitions in a natural language, for example. Finally, an ontology is shared, because it describes a consensual knowledge, which is used by more than one individual and accepted by a group.

According to Noy [10], there are some reasons that can define the importance of ontologies:

- Sharing common understanding of the structure of information among people and software agents;
- Allowing the reuse of domain knowledge;
- Obtaining explicit domain understanding;
- Separating domain knowledge and operational knowledge;
- Analyzing domain knowledge.

Sharing the common understanding of the information structure among people and software agents is one of the most common goals for the development of ontologies [8]. From the definition of domain knowledge reuse, it is possible that other applications might reuse it. Furthermore, a larger ontology can be built from the integration of a group of other ontologies.

It is also crucial to provide the understanding of an explicit domain. Therefore, an explanation and better maintenance of the terms that comprise this domain become possible. Another relevant characteristic is linked to the possibility of making inferences about the information represented in the domain, through concepts, relationships and other characteristics of the domain that have been defined and implemented.

Separating the domain knowledge from the operational knowledge implies that the ontology, which represents domain knowledge, is represented separately from the application that uses this ontology. Thereby, different applications can use the same ontology and, thus, obtain the same understanding of this ontology.

Finally, it should be possible to perform an analysis of domain knowledge. This analysis is important to verify whether the ontology meets the needs identified for the representation of a given domain. Furthermore, it is important to ascertain whether changes in the structure can be effected and thereby increase the possibility of the reuse of existing ontologies.

### 2.2. Middleware

Middleware is a distributed software layer, or platform, located between the operating system and the application, which enables communication between distributed applications by abstracting the complexity and heterogeneity of environments [11,12]. The middleware must provide high-level primitives that simplify the development of distributed applications. Similarly to the network protocol stack, it can be decomposed into multiple layers [11]:

- Infrastructure: This layer abstracts the peculiarities of the operating system, facilitating the development of network applications; furthermore, it encapsulates the operating system’s native mechanisms;
- Distribution: Allows clients to develop and integrate remote applications in a transparent manner, abstracting location, programming languages, operating systems, communication protocols and also the hardware used;
- Common services: Define reusable high-level and independent services of the application domain; these services enable application developers to only focus on business logic, for instance security and transaction services;
- Specific services: They are services required by certain application domains; these domains may be, for example, telecommunications, electronic commerce and mobile computing; since the services offered by this layer incorporate the knowledge of a domain, they allow an increase in quality and reduce the effort and the life cycle required for the development of certain types of applications.

The use of middleware significantly facilitates the task of distributed application developers. For middleware developers, the middleware is seen as a collection of distributed services, or middleware services, which take the primary responsibility to communicate distributed applications and hide from them the network low-level mechanisms. The middleware often provides additional services, such as security, transactions, names and events, which “adds” value to the communication between distributed applications.

The services offered by the middleware should [13]: meet a wide variety of applications to be implemented in order to enable execution on multiple platforms, enable remote access to other services or applications, support a standard protocol (ideally) and support a standard API, among others. Services should be transparent with respect to the standard API, that is application developers can use a new service without the need to modify it.

The traditional middleware systems require a large amount of memory and high processing power from the hardware, which makes them unsuitable for use in WSNs [14,15]. Consequently, a solution for WSN middleware should consider specific characteristics of WSN applications in order to extend the lifetime of these networks. Therefore, a middleware project for WSNs has the following challenges [2]: power and limited resources management, heterogeneity, scalability, dynamic network topology, data mobility and aggregation.

### 2.3. TinyOS and nesC

The development of operating systems for WSN nodes must be designed to save as much energy as possible. Furthermore, it should be efficient in terms of memory consumption and processing and be agile enough to allow multiple applications to simultaneously use the communication resources. Therefore, TinyOS [16] is a widely-used open-source operating system to run on devices with low computational power and limited resources, such as WSNs, and implemented in the nesC programming language [17].

nesC has been developed with initial challenges, such as robustness, limited availability of resources, different implementations of the same service, evolution of the hardware and adaptability to the requirements of applications. The main feature of nesC is a holistic view of the system. WSN applications are highly dependent on hardware, and each node runs only one application at a time [17].

nesC is a component-oriented language with an event-based execution model. One of the greatest advantages of this component-oriented model is the fact that the developer can build an application using a set of existing components and adding extra code, when needed, to run the application. Then, instead of being a general purpose operating system, TinyOS behaves as a framework, avoiding the use of components that are not necessary to run the application.

Another remarkable feature of nesC is the support for TinyOS concurrency models, performing optimization and detection of data concurrency at compile time. This is a simplified concurrency model, which allows concurrency with low overload, unlike the concurrency model based on a thread, in which the stack of threads requires large memory consumption. However, as any competing system, race conditions, starvation, deadlock and non-determinism are possible sources of bugs. Trying to avoid these competition concerns, TinyOS attempts to identify and ensure the absence of race conditions at compile time. Besides the detection of race conditions, the compiler performs the creation of components statically and dead code elimination.

A nesC interface is bi-directional and used to communicate components. The interface specifies a set of functions called commands or events, where commands are implemented by the event provider interfaces, while events are implemented by user interfaces. A task is another kind of function present in the component. It is a special type of function with no return and no arguments, and it works independently of a control, as opposed to events and commands. In this way, interactions between components can be very complex, and typically, a component registers interest in some event that is signaled when it occurs.

## 3. Related Work

Several studies have used ontology merging theories and the Semantic Web in WSNs. However, little attention has been paid to the semantic representation of WSNs for power consumption. The idea of introducing Semantic Web concepts and creating information systems based on ontology for WSN was introduced by Avancha *et al.* in [18]. In this work, the authors classify important characteristics of a WSN node into an ontology, which describes its functionality and current status.

The work proposed by Avancha *et al.* has provided the basis for several works merging WSNs and semantics, but the majority uses ontologies to provide data with semantics without the concern of reusing these data for improvements in the network. In Eid *et al.* [19] and Huang *et al.* [20], ontologies are defined in order to describe concepts and relationships of WSNs from the network data and to improve the accuracy of queries. In [21], data from a WSN are used for fire hazard rating applying Semantic Web technologies for the processing of data streams, which allow domain experts to specify and adjust the rules to calculate fire rates. As for [22], the Semantic Web is used in WSNs for precision farming applications. That therefore enabled interoperability among different standardized sensing systems.

There are also several solutions that are aimed at reducing power consumption in WSNs. Samuel *et al.* in [4] use clustering techniques to reduce the amount of data transmission between WSN nodes, which are in charge of the greatest power consumption. To do so, the WSN node with greater connectivity is calculated so as to determine which one will be the cluster head. Although there is a concern about power consumption, processing for selecting the cluster head is done by the WSNs, which also generates power consumption in the process.

As for [23], Dâmaso *et al*. used an approach that evaluates the power consumption in WSNs using simulation models. With the result, application developers have enough predictions to choose the module that uses less power consumption for their applications as they are being developed.

Anastasi *et al.*’s work in [3] demonstrates what energy expenditure is like in various different parts of a WSN node and discusses the main forms to conserve energy in WSNs. They also feature a comprehensive taxonomy of energy conservation systems.

In the work of Andreou *et al.* [5], a framework that optimizes network efficiency has been developed, combining load balancing components, minimizing the reception of inefficient data and a query processing module, which uses semantic processing. Even with an improvement in power consumption reductions, all of the steps for energy saving are processed by the WSN nodes.

Although Molina *et al.* [24] use a semantic middleware for wireless sensor networks, its use is centric in a specific context for monitoring physical parameters on a person. In SITRUS, its use is more generic and focused on reconfiguration, with the possibility of many different types of applications taking advantage of the infrastructure, including communication with each other.

Unlike previous works, the SITRUS infrastructure uses a semantic database for queries and decision-making on the reconfiguration of WSN nodes, with data transmitted by RAMSES and managed by SIP (Semantic Information Processing). This approach aims to reconfigure WSNs in an automated manner in order to significantly reduce the power consumption.

## 4. SITRUS

This section presents SITRUS, a semantic infrastructure for WSNs that improves power consumption and shares information between different networks. SITRUS consists of a reconfiguration-aware middleware, called RAMSES (Reconfiguration Aware Middleware for wireless SEnsor networkS), and a SIP module. We initially present a SITRUS overview, through the most important characteristics of the proposed solution, followed by the semantic information processing module with all of its layers and the ontology used. Finally, we present the RAMSES middleware along with its components.

### 4.1. Overview

As mentioned before, WSNs have limited resources, and the power consumed by nodes is one of the main concerns about them. As human intervention to change its batteries is unusual, some WSN nodes can be overloaded and have their energy drained, leaving some network areas blind. Therefore, the best practice is to maintain the network as active for as long as possible and to leave it under its own control.

SITRUS reduces the power consumption by reconfiguring and sharing data between different applications and networks. This infrastructure is not only able to deal with the heterogeneity of data and applications, but it also gives data from WSNs a formal and unambiguous semantics.

The key point of SITRUS is its reconfiguration scheme. Since it is difficult to change the batteries of WSN nodes, an approach has been developed in which WSN nodes themselves decide at runtime the proper moment to alter their behavior so as to save energy. Based on [25] and adapted to WSNs, such a reconfiguration can be done in three different ways:

- Application level: Changing parameters that influence the application execution, such as the periodic sampling of the WSN node;
- Middleware level: Modification of the middleware internal algorithms or some services, such as a routing algorithm; and
- Network level: Putting some WSN nodes to sleep in order to maintain the WSN with the smallest number of WSN nodes.

In order to support all mechanisms previously mentioned, SITRUS has been divided into two distinct parts: a semantic information processing (SIP) and a message-oriented middleware for WSN (RAMSES). Both parts feed the semantic database, generate information about the network and the WSN nodes and give support for the mechanism of reconfiguration. Additionally, using this semantic database, we may have queries that utilize a formal and standardized information model without the need to ask frequent questions of the network.

**Figure 1:**
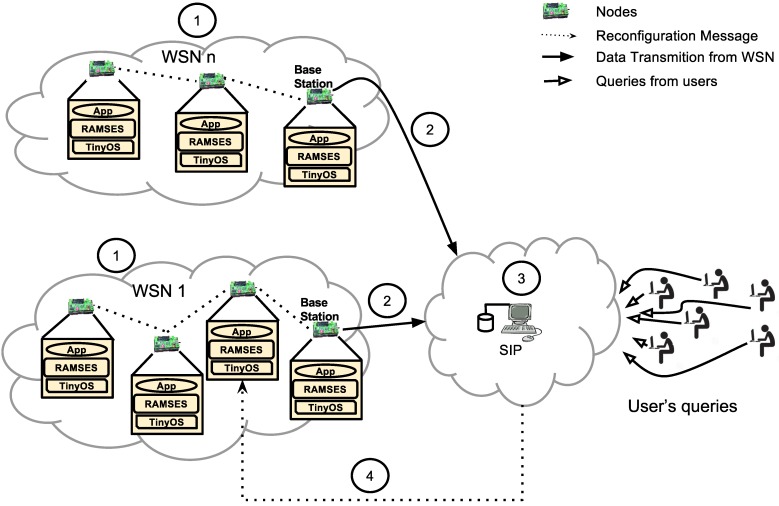
Usage scenario of SITRUS (semantic infrastructure for wireless sensor networks).

Figure 1 depicts a usage scenario of SITRUS. Each step of this scenario has been numbered to facilitate its understanding:

- Step 1: The WSN nodes, which may even been in different networks and running different applications, should be running the RAMSES middleware. In this way, we have a common platform that hides the system’s heterogeneity and allows the reconfiguration of WSN nodes. All data generated are sent to their respective base stations;
- Step 2: All data collected by the base station is forwarded to SIP;
- Step 3: The data collected is processed, categorized and stored in a semantic database within the SIP. Users that need to access data on a particular application or WSN execute their queries on SIP, saving WSN energy by not using the WSN directly and having more accurate information;
- Step 4: In addition to storing data, the SIP monitors the behavior of the network and individual WSN nodes. In case a node is either overloaded or sending redundant information in a given period of time, SIP sends to the WSN node a message of reconfiguration. RAMSES transport this message and triggers the reconfiguration of the WSN node.

Energy saving is provided by the use of the communication model adopted, parametric reconfiguration service execution and usage of low power consumption mechanisms, developed for the RAMSES. In SIP, the power saving relies on the usage of the semantic database as a way to support the data queries on WSNs, in order to avoid unnecessary messages to WSNs. Thus, SIP performs all of the decision-making process on the reconfiguration mechanisms of the WSN nodes.

This way, it can be said that: the WSN internal mechanisms change at runtime; its implementation aims at low power consumption; and part of the data processing being made by SIP helps with lowering the power usage.

### 4.2. Architecture

**Figure 2:**
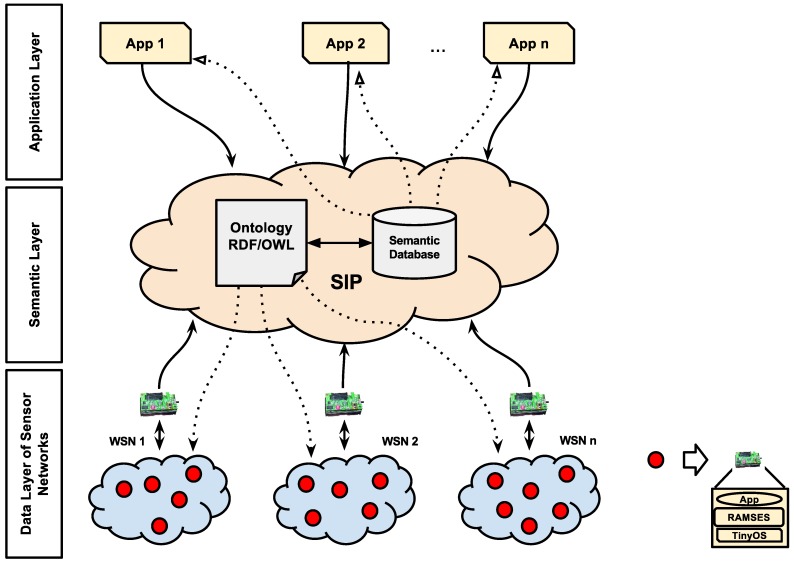
SITRUS architecture.

From the use of ontologies, the proposed architecture allows machines to have knowledge of WSNs, where all data are processed and adapted to the WSN node application requirements. As presented in Figure 2, the architecture is divided into three layers, as follows:

#### 4.2.1. Data Layer of the Sensor Networks

The data layer of sensor networks consists of heterogeneous WSNs formed by distinct sensor nodes with different hardware and applications. The data from each WSN are grouped in their base stations and transmitted to the semantic layer, so they can be processed by it. Furthermore, the semantic layer provides support for parametric service reconfiguration of the data layer of sensor networks, by sending reconfiguration messages to WSN nodes.

The only restriction for this layer is that all WSN nodes adapt their legacy software or build new applications making use of RAMSES, thus ensuring the same communication mechanism between remote components and the use of middleware services in a transparent way.

In this layer, energy saving occurs by the use of the communication model adopted by sensor nodes through the execution of a parametric reconfiguration service and the use of low power mechanisms, developed for RAMSES.

#### 4.2.2. Semantic Layer

Already with the data from the WSN nodes available through its base station, the data are instantiated in an ontology, categorized and stored in a semantic database. Therefore, the second architecture layer refers to the semantic layer for WSN.

In addition to the WSNs sharing the use of RAMSES, they also share the use of the ontology to map their data, which facilitates the development to generate knowledge among different WSNs, since the semantic database and the concepts mapped are the same for all WSNs.

In this layer, energy saving occurs by the use of the semantic database as a way to support the WSN queries, thus avoiding unnecessary messages in WSNs. In addition, this layer performs all processing for the decision-making process of the sensor node reconfiguration.

#### 4.2.3. Application Layer

The application layer consists of different applications that request information of the WSN nodes through data found in the semantic database.

As presented in this architecture, no query is performed directly on any WSN, only on the semantic database. In addition, the result of a query can be a combination of more than one WSN at the same time, because the data of the semantic layer is the result of the combination of several WSNs and their sensing.

The next subsections present the details of the SIP and RAMSES.

### 4.3. Semantic Information Processing Module

SIP is a module that works as an intermediary between the WSNs and the semantics of the infrastructure. To accomplish such a purpose, each WSN node has an instance of RAMSES executing on it. Consequently, the communication from WSN nodes to SIP is based on the same distribution mechanism guaranteed by RAMSES, and all data from WSNs are categorized, organized and appropriately instantiated in an ontology by SIP.

SIP also aims to generate a semantic database to store information concerning WSNs and WSN nodes based on the ontology. Furthermore, it runs several steps for the decision-making of WSN nodes’ reconfiguration and provides access to WSN data in an easier way. Therefore, all queries on the WSN return a piece of data processed by the semantic database, and not directly from the WSN, thus obtaining more accurate and unambiguous data.

On that account, SIP has four basic functions, which are as follows:

1. Receive data from WSNs: By using a common API, all data from the WSNs are sent to their respective base stations and contain information about the sensing of the environment, as well as information regarding the state of the network;
2. Instantiate the ontology data: Data obtained from the WSNs are categorized and instantiated by ontologies. Therefore, we can infer new data and search for data relationships within the same WSN or between different networks. All instantiated data serve to form the knowledge database of the WSN;
3. Process queries: Having the knowledge database populated, queries over the network no longer need to be made directly on the WSN. All processed queries have the populated knowledge database as data source, thereby avoiding ambiguities or incorrect answers;
4. Send reconfiguration messages: SIP also performs automated queries about the status of the WSNs and their nodes. With this, we can check if a particular WSN node has reached a predetermined threshold, for instance a WSN node that is either overloaded or sending redundant information in a given period of time. In this case, a reconfiguration message is sent to this WSN node to normalize its behavior.

**Figure 3:**
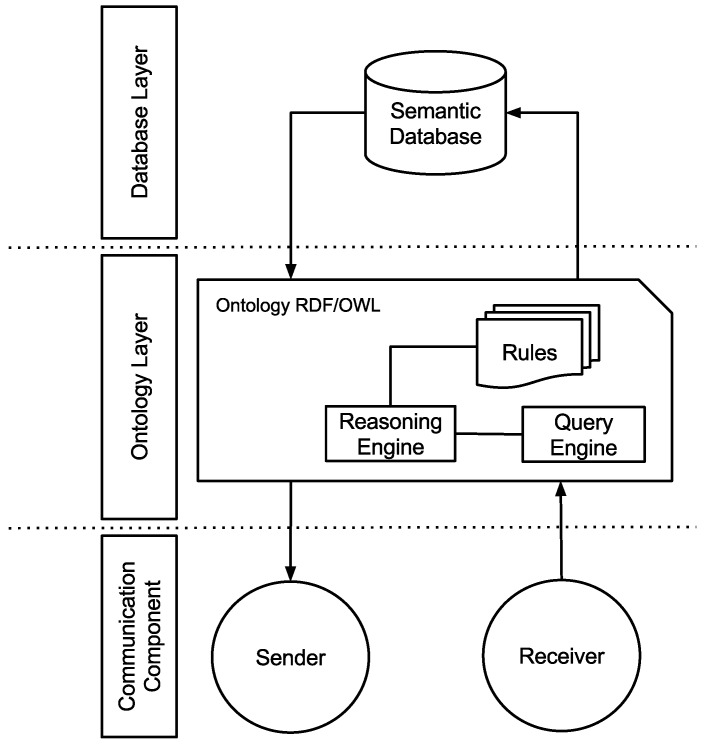
The SIP (Semantic Information Processing) architecture.

As presented in Figure 3, the SIP architecture is divided as follows. The communication component is in charge of the communication with the WSNs. It receives and sends reconfiguration messages to the WSN nodes. As all data are processed by the proposed ontology (see Section 4.3.1), this component communicates directly with the ontology layer. The ontology layer is responsible for handling the proposed ontology, *i.e*., for the instantiation of the data originated from the communication component. This layer integrates new knowledge from existing knowledge, by using deductive reasoning, rules and relationships by the reasoning engine.

Thus, the rules, automatic procedures to generate new relationships based on the data and on some additional information, are not limited to information related only to the deductive reasoning of your classes and relationships, but also by a combination of terms and their relationships, generating new data. Besides inferring new information derived from a knowledge of the reasoning engine, it also has the ability to respond to various types of queries from the queries engine, based on direct knowledge or on computed inferences.

As mentioned before, an advantage of the use of ontologies is the possibility of extracting new data from the existing ones, through rules and relationships. Therefore, one can make queries, including, as new data, the ones obtained by inference. For example, if a WSN Node A sends messages to a WSN Node B, and the B WSN node sends messages to a C WSN node, by inference, we can define that there is a path from the WSN Node A to the WSN Node C.

#### 4.3.1. Proposed Ontology

The use of ontology enriches the data of the WSN by the association of its meaning. Thus, an ontology for the SITRUS infrastructure has been developed and proposes a model of knowledge for the elements of the WSN nodes, data acquisition and energy, which are key concepts to represent a WSN. The developed ontology is generic for elements of any type of WSN. Therefore, it maps concepts independently of the application in WSN nodes. Besides, changes in communication mechanisms or RAMSES services will not change the concepts developed for the ontology.

These concepts were mapped into classes with properties assigned to them. The proposed ontology contains a hierarchy that defines a WSN, describing its features and capabilities.

**Figure 4:**
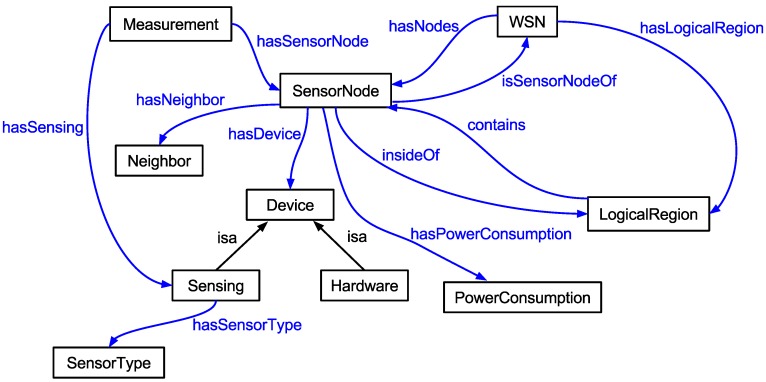
The classes of the ontology proposed.

As shown in Figure 4, the ontology determines the relationships and classes that define a WSN node. The class *SensorNode* includes some basic information about WSN nodes, like *NodeID*, the identification of the WSN node. This class represents the identity of a node in a specific WSN through the relationship with the class *WSN* from the transitive and inverse properties *hasNodes* and *isSensorNodeOf*. Consequently, we can have different networks with identical node identifiers without causing ambiguity.

As we can observe from the relationships represented by arrows, we have support to automatically set the logical regions from the neighboring nodes, which are represented by the class *Neighbor*. A logical region is a region where the data collection, processing and transmission from the WSN nodes are performed by each region, resulting in considerable savings of resources and, consequently, increasing the network lifetime.

A neighbor can be represented as a WSN node that is part of the same coverage area for sending and receiving messages from other WSN nodes. This ontology rule is presented next and defines the existence of a path between WSN Nodes A, B and C:
  isNeighbor(?A, ?B) ^ isNeighbor(?B, ?C) -> isReachable(?A, ?C)  

Each type of WSN node, represented by class *SensorNode*, has components with different power consumption. The manufacturer’s datasheets, represented by class *Device*, serve as a parameter to fill in the values of power consumption by hardware from WSN nodes and thereby to estimate the power consumption. The class *PowerConsumption* is responsible for such mapping.

The class *PowerConsumption* contains information about the power consumption of the WSN nodes. As there is a relationship between this class and the class *SensorNode*, *hasPowerConsumption*, we can associate the individual consumption of each node (in Joules), as well as the total power consumption of a given network or logical region.

The class *Measurement* maps data from WSN nodes and their type of sensing directly, but indirectly, through its relationships with other classes, can map the data on the type of hardware, neighbors, logical region and what WSN a WSN node is a part of, and so on.

In addition, important information is added to the class *Measurement*: the *Time* field. It is used to identify the moment when an instance of the ontology was created. Thus, a query can be performed, for example using only the data from the last five minutes of a given WSN node. The one who defines the relevance of time for a given query is the user and not the SIP, because time requirements may be different among users.

With all data instantiated and categorized by the proposed ontology, using their properties and rules, we have enough information for the decision-making process, which occurs in the semantic layer of the SITRUS architecture. In addition, the decision-making process may vary, depending on the application needs, leaving the architecture generic enough for many different situations.

### 4.4. RAMSES

As mentioned before, RAMSES is a message-oriented middleware that is part of the SITRUS infrastructure. RAMSES is responsible for transporting the data generated by the WSN to the SIP and the service management of WSN nodes, besides taking care of the reconfiguration of the latter. While the SIP determines when the reconfiguration process occurs, it is RAMSES’s reconfiguration service that performs the needed actions, based on messages sent by the SIP.

**Figure 5:**
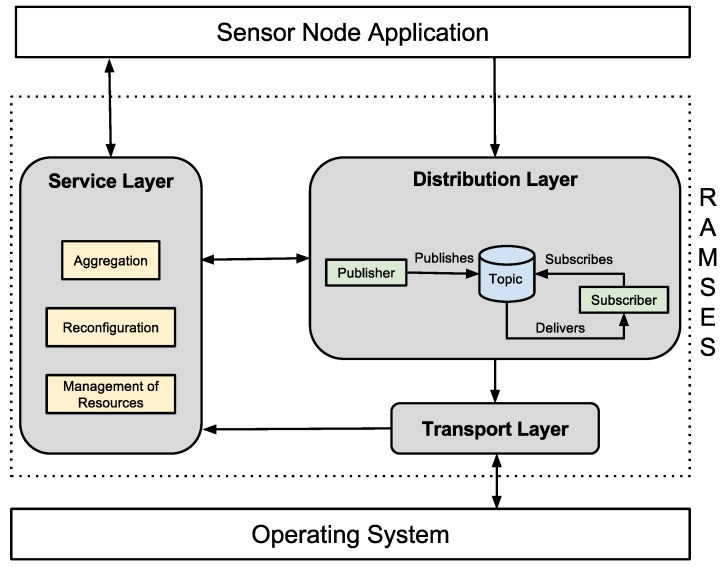
RAMSES architecture.

Unlike traditional middleware platforms, which contain several services, WSN middleware systems have only a small number of services. Hence, RAMSES provides the aggregation, reconfiguration and management of resource services. The architecture of RAMSES has been defined as shown in Figure 5. According to this figure, RAMSES is positioned above the operating system, encapsulating its internal details and providing high-level services for the application of the WSN node.

The transport layer handles the details of communication provided by the operating system, thereby encapsulating its complexity for the applications. As it is the layer that handles communication, it is responsible for transmitting and receiving SIP messages. Every time a new message is sent from the SIP to a WSN node, this message is forwarded from the transport layer to the service layer, so that it can be processed by the reconfiguration service.

The distribution layer implements the publish/subscribe communication model, by publishing messages and mediating the communication between other middleware services. Furthermore, it allows transparent communication between remote clients, abstracting the location, communication protocol and hardware used.

The distribution model of RAMSES is based on an asynchronous message-sending model [26,27], which is more adequate for the information dissemination model required by WSNs applications. In this type of communication, a publisher publishes the messages that are sent to one or more subscribers. Asynchronous communication is the main advantage of this model of communication in the context of *ad hoc* and diffuse environments, such as WSNs. Additionally, all messages are associated with topics [14]. As a result, only subscribers receive the messages associated with the exact topic to which they have subscribed.

The model choice to publish/subscribe is due to the fact that WSN applications are based essentially on events (event-driven), which suggests a more adequate communication model than the traditional request/response model, which is synchronous. This way, the more adequate communication model dissemination of information [28,29] is the asynchronous model, required for WSN applications [26].

The amount of information generated in a physically-dispersed network with uncountable WSN nodes sharing data is huge. In this scenario, some WSN nodes and some base stations are not always active and communicating in the network at the same time. This way, the synchronous communication, based on the request/response model, is not always adequate to meet such requirements.

The last layer, namely the service layer, is responsible for providing some services, such as aggregation, reconfiguration and management of resource services. The aggregation service, which is defined for each WSN node, allows the reduction of data volume in the network, by aggregating the data using a given function, such as the average, mode or median. Each WSN node waits for a predetermined amount of messages and gathers all data received during this period. Afterwards, it sends these data through the transport layer. The implementation of this service in RAMSES is parametrized, which leaves the reconfiguration service with the amount of data to be aggregated and the aggregation function to be adopted.

An important service provided by RAMSES is the reconfiguration service. It is a parametric service that changes the behavior of a WSN node at runtime to save energy. All of its actions are always seeking a better use of energy and are based on messages sent by the SIP. Hence, the whole process of decision-making to decide when to change certain behaviors stays outside the WSN, but the behavior change of a WSN node, based on this message, is handled by the reconfiguration service.

All of these process are managed by the service for the management of resources.

### 4.5. Implementation

In this section, we present the implementation details of major parts of SIP and RAMSES, as well as some considerations about it.

#### 4.5.1. SIP

SIP was implemented using the Protégé tool [10] for ontology development and the Jena framework [30] for manipulating instances in Java. Jena provides an API to read and write data in Resource Description Framework (RDF) [31] or Web Ontology Language (OWL) [32] graphs, which are represented as some abstract models of knowledge that we wish to map, in this case WSNs. Jena also provides a mechanism for queries and a reasoning engine to perform inference over the graphs’ data. With this, the WSN data can be mapped and categorized based on their semantic definitions, thus building the semantic database and giving support to other layers.

As shown by the SIP architecture in Figure 3, the communication component is responsible for the communication and acquisition of data. Furthermore, it is also responsible for sending the received data to the ontology layer and for receiving a reconfiguration message from the latter, which will then be sent to the WSN. The code for sending messages to the reconfiguration of a particular WSN node is presented as follows:

 **public void** sendMessageToSensors(**int** sensorNode) {
  **try** {
   mote.send(sensorNode, getMessage());
  } **catch** (IOException ex) {
   Logger.getLogger(DataManager.**class**.getName()).
        log(Level.SEVERE, **null**, ex);
  }
 }


The ontology layer is responsible for the implementation and manipulation of the ontology in Java. This implementation includes interfaces and methods that handle all of the properties, rules, axioms and relationships established for all classes of the proposed ontology. The ontology layer is also responsible for the use of the inference engine in order to infer new data from the collected data, their relationships, properties and rules. Moreover, it is responsible for the composition and handling of queries related to the data stored in the semantic database. Therefore, decision-making concerning WSN nodes happens according to such queried data.

The query code used in the implementation is depicted in the following.

 **Select** ?x1 ?value
 **Where** {
  ?x1 ?y1 or:Measuring .
  ?x1 or:value ?value .
  ?x1 or:hasSensorNode or:SensorNode_
 }


This query, implemented in SPARQL [33], is used to determine the measurement data of a WSN node. A result like this query is used to make decisions concerning WSN nodes. SPARQL is a query language for databases that is able to retrieve and manipulate data stored in RDF format. It can be used to express queries across different data sources, whether the data are stored natively as RDF or viewed as RDF via an application.

#### 4.5.2. RAMSES

RAMSES is based on the TinyOS components model [16] and was implemented in the nesC programming language [17] (see Section 2.3). Its implementation is driven using low power listening and parametric reconfiguration at runtime. Power management is aided by the use of the low power listening (LPL) interface, which is provided by the TinyOS itself. The LPL interface enables us to more efficiently manage the use of radio operations that only leave the antenna connected when some activity on the communication channel is detected.

The implementation is based on the clear channel assessments method (CCA) [34], to determine if there is a nearby transmitter, checking that the communication channel is clear, thus avoiding collisions. This allows the receiver to turn the radio on and to determine that there are no transmitters in a very short period of time, rather than leaving the radio on long enough to receive a complete package.

Leaving the antenna connected, even though it is not used, consumes energy. Thus, using a method that is concerned with the period of time that an antenna must be turned on assists greatly in reducing energy consumption.

The nesC code used to start an application in a WSN node using the low power listening interface is presented as follows:

 event void Boot.booted() {
  call LowPowerListening.setLocalWakeupInterval(INTERVAL);
  call RadioControl.start();
  call MainTimer.startPeriodic(INTERVAL);
  call Middleware.createTopic("App");
 }


**Figure 6:**
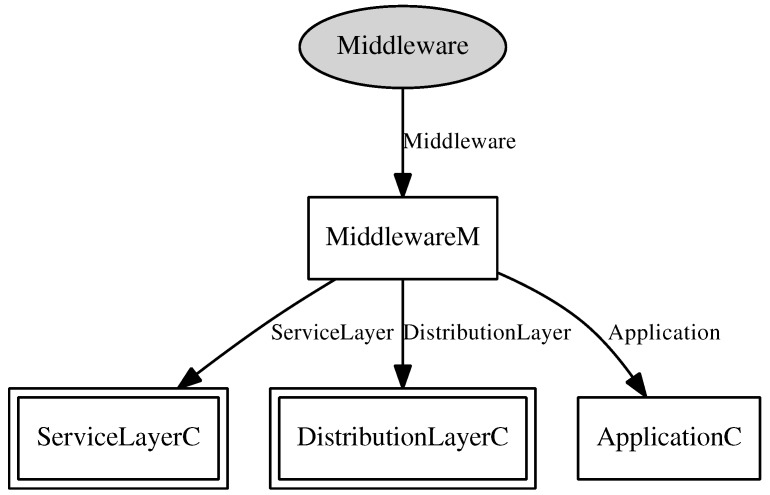
Middleware component.

**Figure 7:**
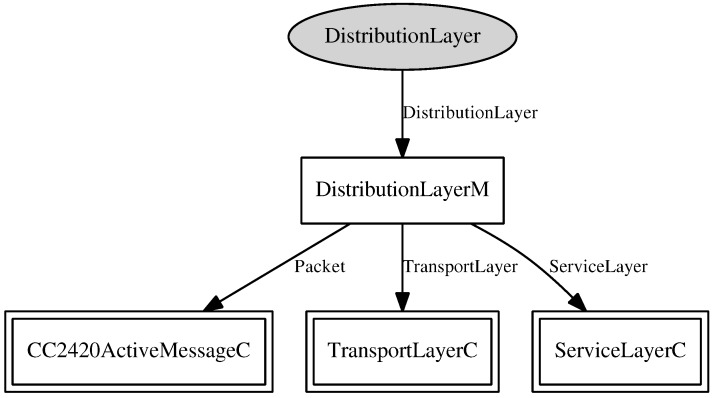
Distribution component.

In the development of RAMSES, the middleware component, a nesC component presented in Figure 6, is the component that comprises the service layer and distribution layer of the RAMSES architecture, presented in Figure 5. This component provides two operations, namely *send* and *createTopic*. The operation *send* is implemented as a command, and it invokes the message sending command from the transport component. As for the operation *createTopic*, it is a call to the command of topic creation from the distribution layer component, presented in Figure 7.

**Figure 8:**
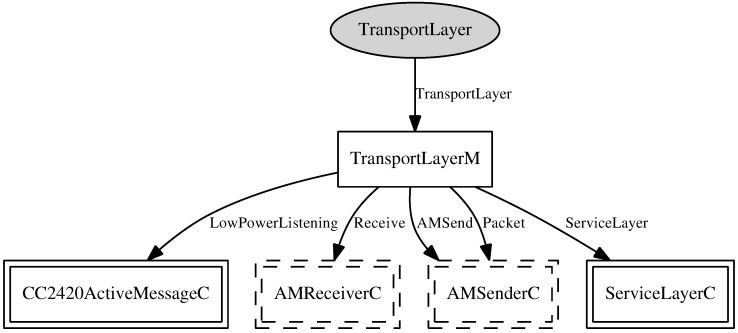
Transport component.

The transport layer component, shown in Figure 8, implements the communication services between remote components, such as WSN nodes, base stations and SIP. It provides an interface with two operations: *send* and *receive*. The *send* command aims to assemble a message that needs to be sent to remote clients. Therefore, a call is made to the module *AMSend* for data to be sent over the network. The *receive* is intended to signal the reception of data by the base station and the WSN node, as well. To accomplish this task, the TinyOS module *AMReceiver* is used.

As for the distribution layer component, presented in Figure 7, this is the component that implements the distribution layer of the RAMSES architecture, providing an interface with three operations:*createTopic*, *publish* and *subscribe*. These operations are responsible for enunciating and maintaining the list of topics provided by the middleware, publishing messages containing data related to a particular topic and defining the message subscribers, respectively. As a way of saving energy, only messages concerning subscribed topics are transmitted.

For a packet to be mounted, have its data read or sent for transmission, the distribution layer component makes use of the *CC2420ActiveMessageC* component in its implementation, that is responsible for the access to a package of data. All of these operations are made utilizing low power usage.

Every time a message is ready to be published, with all of its information filled, it is executed the *send* command from the transport layer component for this message transmission. This way, all data acquisition and transmission operations are on a different layer than the creation, signature and topic and messages publish operations, so that each layer has its responsibilities well defined.

Before publishing a message, some metadata need to be filled, for instance the WSN node identification, the WSN node current sampling rate and the amount of aggregated data per message. These metadata are used by the SIP for the processing of the current state of a WSN node.

**Figure 9:**
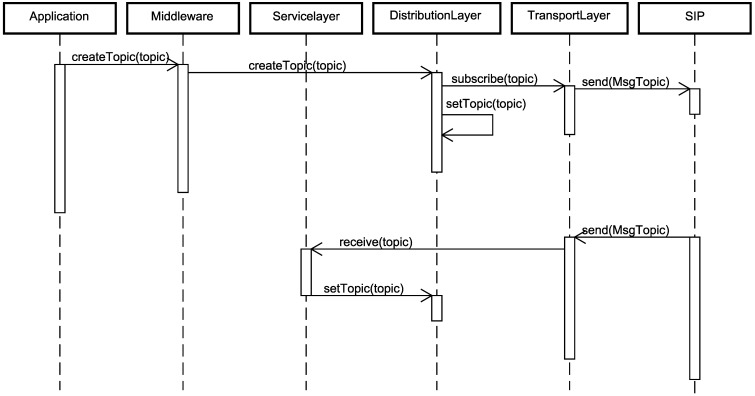
Sequence diagram for the signature and announcing of a topic.

The sequence diagram in Figure 9 shows the interaction between several layers of the RAMSES middleware and the SIP for the signature and announce of WSN node topics. By default, each application is associated with only one topic, which can also be changed during the application execution. Thereby, from the creation of the topic by the WSN node, it will immediately be signing its participation.

The application announces to the middleware component the sensing operation (temperature, humidity, light, among others) that is being performed and sends it, as a parameter, through the *createTopic* method, which in its turn forwards the message to the distribution layer component. The announcement of topics is not directed to other WSN nodes, but only to the SIP, which stores this information in its semantic database. To accomplish such a task, the distribution layer component executes the *subscribe* method, with the topic as a parameter, for the transport layer component, so that the message can be transmitted to the SIP.

The interaction in the bottom part of Figure 9 refers to the occurrence of topic changes by the SIP or the non-creation of a topic by the application being executed in the WSN node. For both cases, the execution is the same. In case the application changes its sensing type, as a requirement of the application itself or through reconfiguration sent by the SIP, its topic also needs to be changed. In this way, the SIP sends a message to the WSN node with the new information on the new topic. This message is received by the transport layer component and forwarded to the service layer component, from the *receive* command. After this stage, a call is made to the *setTopic* command from the distribution layer component, and with that, from the new topic, which was sent as a parameter, the WSN node has its data updated with the new topic associated with it.

**Figure 10:**
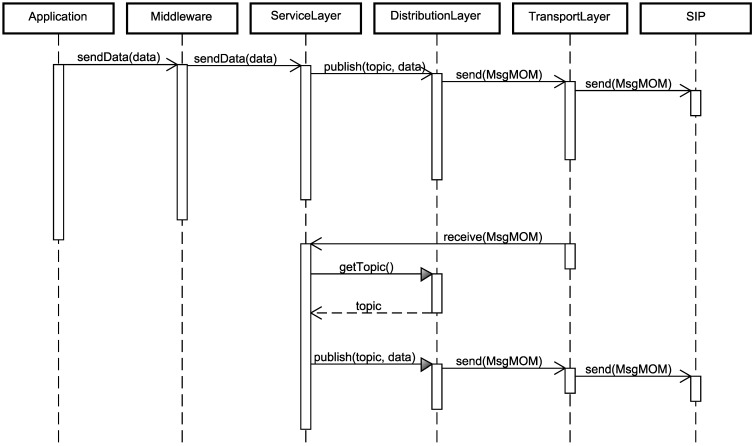
Sequence diagram for a message publication.

Figure 10 shows how the monitored data by the WSN nodes are published in the network until their arrival at the SIP. The application periodically collects sensing data and sends these to the middleware component through the *sendData* command. Therewith, data are forwarded to the service layer component, for its data to be processed by the existent services, for instance the aggregation data service. With these data processed and ready to be sent, the *publish* command is executed, and the message follows its flow to the SIP, from the base station.

Finally, the interaction at the bottom part of Figure 10 occurs when a message arrives at a WSN node from another. The transport layer component, as soon as it receives a message, forwards it to the service layer component, through the *receive* command. At this component, a comparison between the received topic by the message and the matching topic of the WSN node is made, through the *getTopic* command of the distribution layer component. In case both belong to the same topic, the message follows its flow with the execution of the *publish* command, until it reaches another WSN node or the SIP, from the base station. In case the WSN node is not associated with the received message, the message is discharged. In this way, unnecessary messages are prevented from being sent through the network.

**Figure 11:**
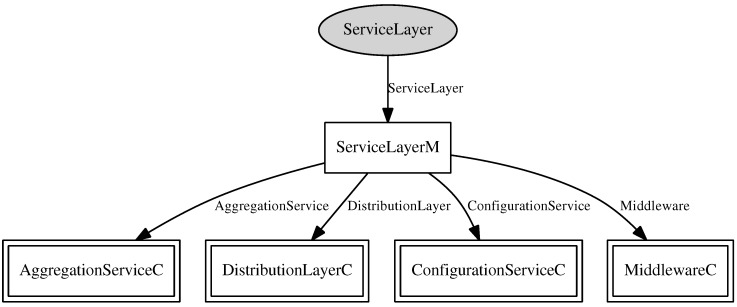
Service component.

Finally, the service layer component, presented in Figure 11, implements the service layer of the proposed RAMSES architecture. It provides an interface with operations, responsible for checking and changing the sampling rate with the *getPeriodicSampling* and *setPeriodicSampling* commands, respectively. It is also responsible for checking and altering data aggregation, with the *getAggregationSize* and *setAggregationSize* commands, and for receiving SIP messages and interpreting its result with the *receiveSIPMessage* commands. This command identifies what type of reconfiguration message has been sent by the SIP and makes the proper call to the corresponding command.

The nesC code used to start the reconfiguration process from the messages received by SIP is presented as follows:

command void ServiceLayer.receiveSIPMessage(application_msg_t* payload) {
 if (payload->sipMessage == 1) { ... }
 
  else if (payload->sipMessage == 2) { ... }
  else if (payload->sipMessage == 3) {
   call ConfigurationService.setPeriodicSampling(payload->sampling);
   call Middleware.changePeriodicSampling(payload->sampling);
 } else if (payload->sipMessage == 4) {
   call ConfigurationService.setAggregationSize(payload->aggregation);
 } else if (payload->sipMessage == 5) {
   call ConfigurationService.setTopic(payload->topic);
}


## 5. Experimental Evaluation

The objective of this experimental evaluation is to evaluate the power consumption and memory usage of WSN applications built atop SITRUS. It briefly introduces the applications being used in the evaluation.

### 5.1. Measurement Procedure

The nesC applications used in the experiments were deployed in a MICAz mote [35] connected to an MTS300 sensor board. These applications were compiled by nesC Version 1.3.4, built on top of TinyOS 2.1.2.

In order to perform the experiments and to efficiently measure the power consumption of a WSN node, the AMALGHMA (advanced measurement algorithms for hardware architecture) [36,37] receives the captured information as an input for calculating the power consumption.

**Figure 12:**
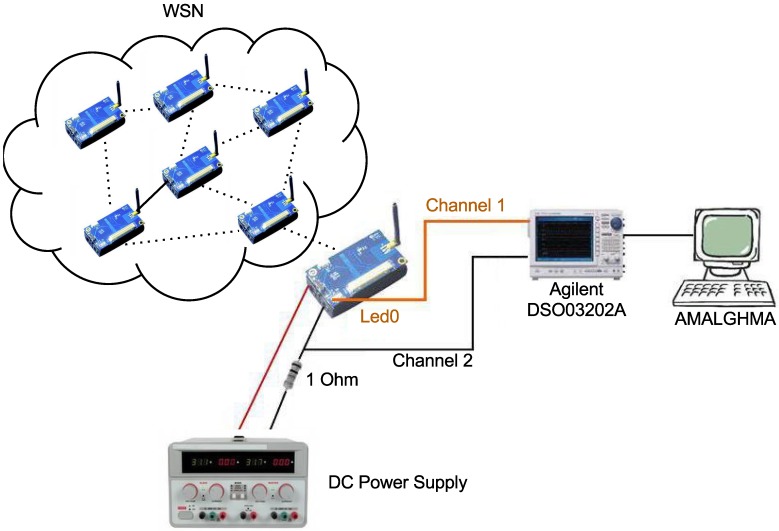
Measurement scheme for power consumption.

The measurement setup is shown in Figure 12 and includes an oscilloscope (Agilent DSO03202A), used to measure the voltage variation of a WSN node, and a DC power supply (iCEL Manaus PS-5000), the function of which is to maintain the same voltage during application execution. A PC connected to the oscilloscope detects the variation of voltage through a one-Ohm resistor (Channel 2). The other channel, Channel 1, captures the activity of the WSN node LEDs, which are turned on or off to signal the beginning or the end of the experiment execution.

As shown in Figure 12, the power consumption calculation is performed by AMALGHMA in only one WSN node, but communication takes place in a WSN with six nodes, all of which are always in the same places in the experiments, where data are sent and transmitted between them.

It is worth observing that a message sent from the SIP to the WSN nodes, as part of the reconfiguration service offered by the RAMSES middleware, controls the experiment’s start and finish. The code used for setting up and conducting an experiment is presented as follows:

 **public void** Measurements(**long** time, **int** periodicSampling) {
  try {
   dataManager.stopAmalghma(); //Leds on
   dataManager.sendMessageToSensors();
   dataManager.setPeriodicSampling(periodicSampling);
   dataManager.sendMessageToSensors();
   Thread.sleep(500); //Time to set the sensors
  
   dataManager.startAmalghma(); //Leds off
   dataManager.sendMessageToSensors();
   Thread.sleep(time);
 
   dataManager.stopAmalghma(); //Leds on
   dataManager.sendMessageToSensors();
  } **catch** (InterruptedException ex) {
   Logger.getLogger(ScenarioDefault.**class**.getName()).
    log(Level.SEVERE, **null**, ex);
  }
 }


Five applications with different characteristics have been used in the measurement. All of them are within the examples of TinyOS [38] applications and have been selected because of their different characteristics of processing, transmitting and environmental monitoring. For each application, three different scenarios have been used to calculate power consumption. The scenarios are as follows:

- Application: The application does not use any power management feature or reconfiguration service. Thus, the application starts the same way as it ends: with no changes in its behavior. This scenario is the control for comparison with other scenarios of the same application.
- Application + RAMSES: The same application of the previous scenario is executed using RAMSES. Therefore, all of the data transmitted between the WSN nodes and the reconfiguration services are transparently guaranteed to the application by the middleware, but no service is executed. Thus, no reconfiguration is carried out. The application is only responsible for collecting and transmitting data from the middleware. To do so, the entire code of the transmission and data acquisition of the original application was replaced by the middleware services. This scenario was designed to evaluate the impact of adding RAMSES in an application.
- Application + SITRUS: In this scenario, SITRUS is used in full. Thus, RAMSES uses its power management based on the low power listening interface, periodically turning the antenna on and off to avoid unnecessary power consumption, but with enough time to detect a carrier and receive or send a data package. The transmitters perform a message delivery by transmitting the full packet over and over for twice the duration of the receiver’s duty cycle period. Transmitting for twice as long increases the probability that the message will be detected by the receiver and allows the receiver to shave off the small amount of time it needs to keep its radio on.
  Furthermore, all of the data are sent to the SIP, so that they are stored in the semantic database, and the decision-making mechanisms for reconfiguration may be used. The reconfiguration service chosen for the applications was to change the sampling rate, from the average of the last samples collected for the same experiment. If the next collected values are the same as the average values, then the sampling rate is increased to avoid data redundancy. When the sampling rate is changed, the power management service is also triggered by changing its time period with the antenna on/off, thus ensuring greater efficiency in power consumption.

For all scenarios, the information sent to the SIP is related to the type of sensing, the identification of the WSN node, the environmental sensing and the sampling rate value. Such information is used to instantiate the ontology and, consequently, to provide a basis for queries on the state of the network and to make decisions concerning WSN nodes.

Finally, applications used in experiments started at the same sampling rate time (250 ms) and had a 4-min period between the start and end of the experiment. Within this time, we have observed behavioral changes in the reconfiguration of WSN nodes. The power consumption is an average of 100 conducted experiments for each adopted approach, with a confidence interval of 95%. All data are summarized in Table 1.

**Table 1:** Elements of the experimental configuration.

WSN Nodes	Confidence Interval	Experiments by Scenario	Experimental Time	Initial Sampling Rate Time
6	95%	100	4 min	250 ms

### 5.2. Results

For each application, the power consumption and memory usage are shown. The memory usage refers to the amount of memory (RAM and ROM) necessary to execute the application in the MICAz mote. In MICAz, the ROM is a 128-KB Flash memory (internal to the ATmega128L microcontroller) for program memory. As for the RAM of the ATmega128 L, it is a 4-KB SRAM, used to store application data.

#### 5.2.1. RadioCountToLeds Application

*RadioCountToLeds* is the simplest application with data transmission that is in the examples of TinyOS [39]. It maintains a counter, which is incremented every 250 ms, transmitting its value in one package whenever its value is updated. In addition to the transmitting count data function, its three LEDs are used to signal the transmission, reception or some kind of error. One of the features of this application is that it does not use the sensing subsystem; therefore, it does not consume energy at this point. This is a useful application to demonstrate how the basic communication mechanisms work in TinyOS.

**Figure 13:**
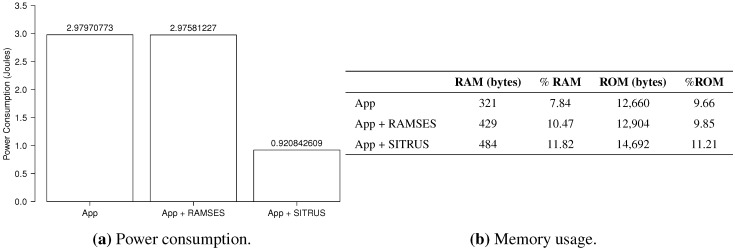
Results of the *RadioCountToLeds* application.

The results of the power consumption of the *RadioCountToLeds* application are shown in Figure 13a. As can be observed, even with the inclusion of RAMSES in the application, the power consumption proved to be pretty much the same, thus showing that it does not affect power consumption that significantly for this type of application.

For the full SITRUS version with all services running, such as the RAMSES’s power management and reconfiguration service of the sample rate at runtime, the reduction of the power consumption was 69.10%. Although it is a simple application, the reduction of the power consumption was significant.

Figure 13b presents the memory usage of the *RadioCountToLeds* application. It uses 7.84% of the RAM and 9.66% of the ROM of the MICAz mote. Meanwhile, the memory usage when RAMSES is used is 10.47% of the RAM and 9.85% of the ROM. In this case, the increase in the memory usage is due to the addition of RAMSES. Finally, with full SITRUS (RAMSES and SIP working together, with all services running), the memory usage reached 11.82% of the RAM and 11.21% of the ROM.

**Figure 14:**
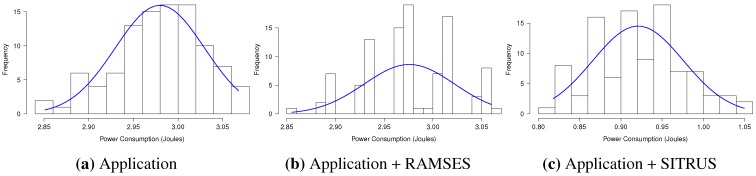
Histogram of the *RadioCountToLeds* application.

The histograms concerning the results of power consumption, obtained for the set of 100 experiments of the *RadioCountToLeds* application, are shown in Figure 14, thereby attesting to the regularity of the results obtained.

#### 5.2.2. RadioSenseToLeds Application

*RadioSenseToLeds* [40] obtains samples of the environment using a basic sensor used by the sensing platform, generating and transmitting the sensing data whenever a new value is collected. In addition, the transmitted or received data, or transmission errors, are flagged by its three LEDs, as in the prior application. Such an application is useful to demonstrate how communication mechanisms, timers and the TinyOS standard sensing subsystem work.

**Figure 15:**
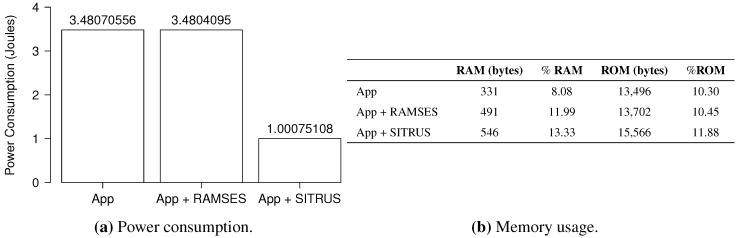
Results of the *RadioSenseToLeds* application.

The power consumption in all scenarios used for the application are summarized in Figure 15a. As can be seen, the original application and its version with RAMSES do not make a significant difference for the power consumption, as the values are almost the same. Therefore, because of its implementation, RAMSES is able to add services for the application at a very low power cost.

The comparison between the original version of the application with its full SITRUS version showed a significant difference: the reduction in power consumption was 71.25%. This result is justified by the use of the low power listening interface implemented by RAMSES, as well as by the alteration in the sampling rate, which is sent by the SIP via a message.

As depicted in Figure 15b, the *RadioSenseToLeds* application uses 8.08% of the RAM and 10.30% of the MICAz mote ROM, with 331 and 13,496 bytes, respectively. As for the memory usage by the RAMSES with the RadioSenseToLeds application, it has been noted that the use of RAM is 11.99% and 10.45% of the ROM, with 491 and 13,702 bytes, respectively. Thus, as occurred in the previous application, this difference happens due to the introduction of RAMSES’s layers in the application.

With respect to the full SITRUS version, the memory usage went to 13.33% of the RAM and 11.88% of the ROM, with values of 546 and 15,566 bytes, respectively.

**Figure 16:**
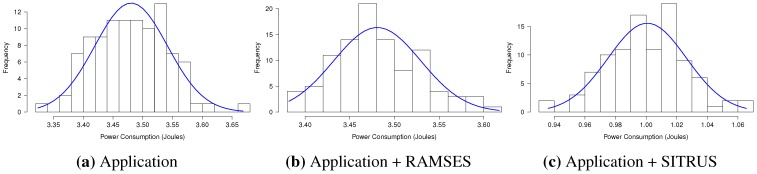
Histogram of the *RadioSenseToLeds* application.

The histograms concerning the results of power consumption, obtained for the set of 100 experiments of the *RadioSenseToLeds* application, are shown in Figure 16, thereby showing the regularity of the results obtained in all of the application scenarios.

#### 5.2.3. Temperature Application

The third application evaluated differs from the previous ones because it uses a specific type of sensing of the MTS300 board, whose function is to measure room temperature. Moreover, in order to return the measure of the sensing in Celsius degrees, an internal conversion processing needs to take place every time a reading is performed. Both the specific sensing and the conversion processing for Celsius are extra sources of power consumption. In addition, transmission signaling and data reception are performed by two of its three available LEDS. This is a useful application to demonstrate the operation of specific sensors, the conversion of sensing data, the communication mechanisms and the timers.

**Figure 17:**
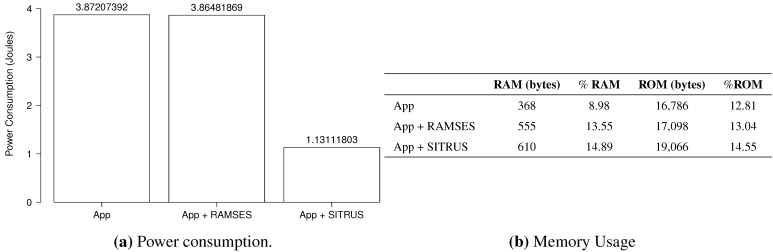
Results of the *Temperature* application.

The evaluation results of the application *Temperature* power consumption are summarized in Figure 17a. Because it uses more internal processing than the previous applications and also because it uses a specific sensing, its results show a higher power consumption compared to previous applications. Still, the same behavior presented in previous applications repeats itself, that is the addition of RAMSES to the original application, with all its layers and available services, has a low power cost if the scenarios are compared.

Concerning the comparison of the original application with its full SITRUS version, it is noticeable that it shows a significant difference in power consumption, with a reduction of 70.79%. This reduction occurs due to the use of the reconfiguration services implemented in RAMSES with decision-making in SIP (by calculating the average of the last samples and by changing the sampling rate in the case of the data redundancy of new samples), in addition to the use of communication at a lower power cost.

As shown in Figure 17b, the original application uses 368 bytes of RAM and 16,786 bytes of ROM; hence, it occupies 8.98% and 12.81% of its total capacity, respectively. With the introduction of layers of RAMSES services in the original application, consumption increased to 555 bytes of RAM and 17,098 of ROM, occupying 13.55% and 13.04% of the entire byte capacity, respectively.

With regard to the full SITRUS version, which uses more components and therefore more memory, RAM consumption in bytes increased to 610 and 19,066 bytes of ROM, totaling 14.98% and 14.55% of its total capacity, respectively. The increase in memory usage is not significant considering the memory that still is available in the mote.

**Figure 18:**
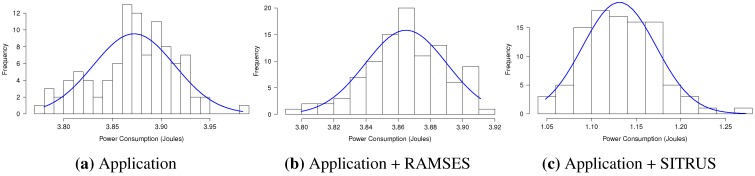
Histogram of the temperature application.

In order to demonstrate the regularity of the data obtained in the 100 experiments for each scenario of the *Temperature* application, the histograms are displayed relating to the results of power consumption in Figure 18.

#### 5.2.4. Oscilloscope Application

The *Oscilloscope* application is one that shows the use of data collection before transmission. To do so, the application *Oscilloscope* [41] periodically performs ambient light sensing, which is another specific sensing of the MTS300 board, but it only transmits data when a set of 10 samples of these readings is completed. Such readings can be received by the base station and have their data aggregated and displayed by the Java application of the same name, which is part of the example applications of TinyOS.

As a major source of power consumption, we have the processing and transmission of the 10-light sensing sample set. This application differs from the others because, instead of sending only one collected sample, it sends a 10-sample set, therefore allowing the aggregation to be performed by another WSN node or by another application. The RAMSES aggregation service is not enabled until the first set of data is to be processed by the SIP, and thus, changes the aggregation rate for 10 samples using the average algorithm.

**Figure 19:**
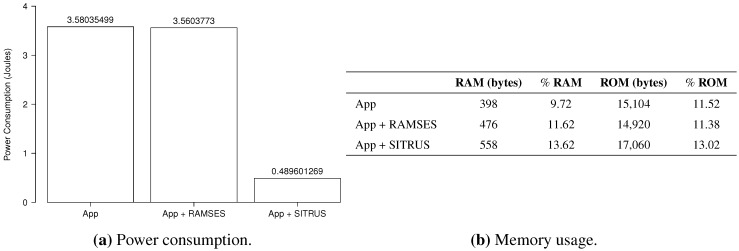
Results of the *Oscilloscope* application.

The results of all power consumption scenarios are summarized in Figure 19a. It is observable that even with the inclusion of RAMSES in the original application, power consumption was almost the same; hence, it shows that, again, RAMSES has a good power performance when compared to the applications ported for it.

As in previous experiments, the application that uses SITRUS achieved a considerable reduction in power consumption, saving 86.33% over the original application. This difference occurs because of the way data are processed. While the original application collects 10 samples and sends the set of collected data, RAMSES aggregates these data in the WSN node itself, thus decreasing the number of bits in a transmission, in addition to the use of data distribution services and the alteration of the sample rate.

As depicted in Figure 19b, the original application uses 398 bytes of RAM and 15,104 bytes of ROM, occupying, respectively, 9.72% and 11.52% of the total capacity of the MICAz mote. With the inclusion of RAMSES in the original application, consumption reached 476 bytes of RAM and 14,920 of ROM, occupying 11.62% and 11.38% of the total byte capacity, respectively.

With regard to the full SITRUS version, RAM consumption in bytes increased to 610 and ROM consumption to 17,060, totaling 13.62% and 13.02% of the full capacity, respectively.

**Figure 20:**
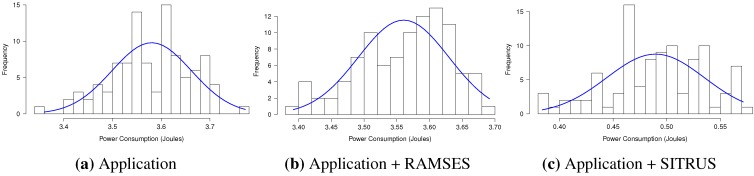
Histogram of the *Oscilloscope* application.

The histograms for the results of power consumption of the *Oscilloscope* application are summarized in Figure 20, in order to demonstrate the regularity of the data obtained in 100 experiments, conducted for each application scenario.

#### 5.2.5. AntiTheft Application

The last evaluated application, *AntiTheft*, is a real environment intrusion monitoring application [42]. In order to detect if there is an intrusion in an environment, the WSN nodes that perform the application identify the level of ambient light. To report an invasion, the WSN nodes blink the red LED as an alert, beep and report the event to other WSN nodes, through network messages.

Both light and sound sensing of the environment are performed by the MTS300 board. To report the data through the network, the *AntiTheft* application uses two different protocols: the *Dissemination* protocol, which disseminates the sensing values for all WSN nodes, and the *Collection* protocol, which allows the WSN nodes to report the sensing values to their root nodes.

**Figure 21:**
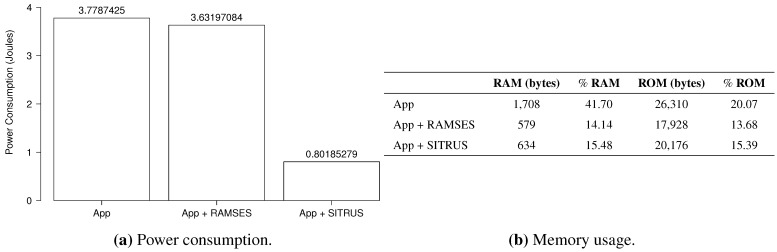
Results of the *AntiTheft* application.

The results of the power consumption evaluation of the *AntiTheft* application are summarized in Figure 21a. Even using network protocols aimed at a better use of energy, the *AntiTheft* application has a high consumption compared to previous applications, due to internal processing and to the use of two different types of sensing. When adding the middleware layer to the original application and exchange communication mechanisms by RAMSES, *i.e.*, *Dissemination* and *Collection* from the original application by the publish/subscribe model from RAMSES, the power consumption has a small reduction.

The comparison of the original application with the version using all SITRUS reveals a significant difference in power consumption, with a reduction of 78.78%. This reduction is due to the distribution model used, by the reconfiguration services implemented in RAMSES with decision-making in the SIP and the use of low power communication mechanisms. Figure 21b presents a memory usage summary for the *AntiTheft* application, with all of the used scenarios in the power usage calculation. The original application consumes 1708 bytes of RAM and 26,310 bytes of ROM, which corresponds to 41.70% and 20.07%, respectively. This high consumption can be explained by the amount of components used for the application development.

With the introduction of new RAMSES components in the original application and the substitution of some others, there was a decrease both in RAM and in ROM. The consumption has become 579 bytes of RAM and 17,928 of ROM, totaling a 14.14% of RAM value and 13.68% of ROM. This decrease occurred due to the replacement of the original application components for the ones used by RAMSES, which demonstrate that even with the introduction of new software layers, both RAM and ROM have a low resource consumption rate.

With full SITRUS usage, we have a rise in memory consumption, in relation to its version with RAMSES, but nevertheless, a lower consumption when compared to the original application. Consumption in bytes has turned to 634 in RAM and 20,176 in ROM, totaling 15.48% and 15.39% of full capacity, respectively.

**Figure 22:**
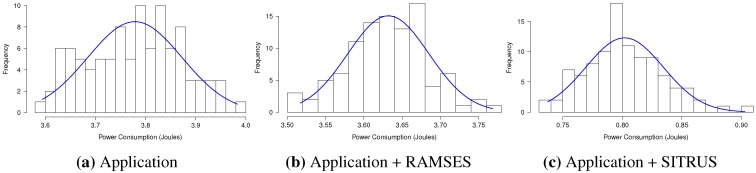
Histogram of the *AntiTheft* application.

Histograms referring to power consumption by the *AntiThef* application results are summarized in Figure 22, in order to demonstrate the regularity of the obtained data at 100 realized experiments for each application scenario.

In all applications, as the application + RAMSES scenario was developed to add an extra software layer with a message-oriented distribution layer, but not using the reconfiguration services involved, then the results with a minor change between the original application and its version with RAMSES were expected, because services that help decrease power consumption were switched off, except the publish/subscribe communication model.

Such experiments have been useful to demonstrate the power consumption of WSN applications built atop SITRUS, which testifies to not only its implementation being efficient, but also that there are benefits of the middleware services used in applications and semantics for the data transmitted over the network. Besides, according to the results of memory usage, it has been observed that the implementation leaves room for new services and implementations.

## 6. Conclusions and Future Works

This paper presented SITRUS, a semantic infrastructure that aims to aid the reduction of power consumption through the middleware RAMSES and SIP, a module for semantic processing of information. With SITRUS, we have data sharing, as well as data semantic enrichment through the use of ontologies.

RAMSES, a message-oriented middleware for WSNs, is in charge of the generation of data for the SIP. It is the part that controls all of the software that runs on WSN nodes and, moreover, looks after the reconfiguration model of the latter. As for SIP, it is a module used for communication between the WSNs and the architecture semantics, which aims to generate the semantic database and to manage the decisions that will form the basis for the reconfiguration of the WSN nodes.

As shown in the experiments carried out to evaluate the power consumption of four different applications, it has been observed that the SITRUS infrastructure appears to be quite effective in its purpose of reducing power consumption.

The main contributions of this paper are: the development of a message-oriented middleware with a reconfiguration service and decision-making outside the WSNs, based on the network’s semantic data; the development of a semantic module to store all knowledge of the applications running on the SITRUS infrastructure, to assist in decision-making and to facilitate information sharing among applications; and finally, the experimental evaluation that shows that such an infrastructure is useful for the reduction of power consumption.

For future works, we intend to perform the evaluation of the power consumption of other services, such as the aggregation one, as well as the assessment of individual modules of the middleware, such as the transport or distribution services. Furthermore, we can expand the use of SIP, determining the logical regions, of particular groups of WSN nodes, from the data sent over the network.
